# Trial registration, publication rate and characteristics in the research field of otology: A cross-sectional study

**DOI:** 10.1371/journal.pone.0219458

**Published:** 2019-07-10

**Authors:** Jan A. A. van Heteren, Isabeau van Beurden, Jeroen P. M. Peters, Adriana L. Smit, Inge Stegeman

**Affiliations:** 1 Department of Otorhinolaryngology and Head & Neck Surgery, University Medical Center Utrecht, Utrecht, The Netherlands; 2 Department of Clinical and Experimental Neuroscience, University Medical Center Utrecht Brain Center, Utrecht University, Utrecht, The Netherlands; Universidad de Antioquia, COLOMBIA

## Abstract

**Objectives:**

To examine 1) the publication rate of registered otology trials in ClinicalTrials.gov, 2) the public availability of the results, 3) the study characteristics associated with publication, and 4) the time to publication after trial completion.

**Background:**

Publication bias, the publication or non-publication of research findings, depending on the nature and direction of results, is accountable for wrong treatment decisions. The extent of publication bias in otology trials has not been evaluated.

**Methods:**

All registered otology trials were extracted from ClinicalTrials.gov with completion date up to December 2015. A search strategy was used to identify corresponding publications up to June 2017, providing at least 18 months to publish the results after trial completion. Characteristics were obtained from ClinicalTrials.gov and corresponding publications. Regression models were used to examine study characteristics associated with publication or non-publication.

**Results:**

From the 419 trials identified on ClinicalTrials.gov, 225 (53.7%) corresponding publications were found in PubMed. Among these, 109 (48.4%) publications were cited on ClinicalTrials.gov and 124 (55.1%) articles reported the National Clinical Trial registry number. For 36 (8.6%) trials, results were only reported in ClinicalTrials.gov. Trials with a biological intervention were more likely to be published than studies involving drugs (odds ratio (OR) 10.41, 95% confidence interval (CI) 1.26–86.22, P = 0.030). Trials funded by industry were less likely to be published (OR 0.46, CI 0.25–0.84, P = 0.011). The median trial duration was 20 months (interquartile range (IQR) 26 months), and median time from trial completion to publication was 24 months (IQR 22 months).

**Conclusion:**

In 37.7% of the registered otology trials the results remained unpublished, even several years after trial completion. With little citations on ClinicalTrials.gov and low reporting of the Clinical Trial registry number, the accessibility is limited. Our findings show that there is room for improvement in accuracy of trial registration and publication of results, in order to diminish publication bias in otology studies.

## Introduction

In an era of evidence based medicine (EBM), clinicians must have access to clear, transparent, and complete information in scientific publications in order to draw conclusions. In 2009, Chalmers and Glasziou made the claim that as much as 85% of research investment is wasted due to a cumulative effect, including the production of biased reports.[1]

Publication bias is the publication or non-publication of research findings, depending on the nature and direction of the results.[2] Selective publication of studies, including delayed publication of completed trials or non-publication, may lead to incomplete or misrepresented evidence, impairing evidence-based clinical practice, undermining guideline recommendations, and can thereby make clinical practice and patient care less effective.[3,4] Previous studies reported that in 23–68% of the registered trials, the results remain unpublished, even several years after trial completion.[3,5–14] Trials that produce negative, inconclusive or non-significant results are less likely to be published or are published significantly later than their positive counterparts.[5,15,16]

Efforts are undertaken to diminish publication bias. First, in 2000, clinical trial registries, such as ClinicalTrials.gov, were established.[5] These registries have the potential to address selective publication by publicly cataloguing clinical trials and promoting trial transparency and accountability.[3] All registered trials receive a unique clinical trial registry number, for example the National Clinical Trial (NCT) number for trials registered in ClinicalTrials.gov. If this number is reported in the article, registered trial information is easy to find.

Second, since 2005, the International Committee of Medical Journal Editors (ICMJE) requires registration of both observational and interventional studies before enrolment of the first patient in a public database as a condition for publication.[17] Consequently, the United States Congress expanded this requirement, only for interventional studies assessing medicines and medical devices, by passing the U.S. Food and Drug Administration Amendments Act (FDAAA), section 801. This mandate requires sponsors or principal investigators of applicable clinical trials to submit results no later than 12 months after completion date, unless legally acceptable reasons for delay are evident. Unfortunately, studies have shown that compliance with the FDAAA 801 provisions is generally poor.[18–20]

Third, from January 2017, the National Institutes of Health (NIH) regulations require that all clinical trials, funded in whole or in part by the NIH, are registered in ClinicalTrials.gov within 21 days after enrolment of the first participant. The results from these trials have to be submitted to ClinicalTrials.gov within one year after trial’s primary completion date.[21] In the European Union, the Clinical Trials Regulation No. 536/2014 will replace the existing Clinical Trials Directive No. 2001/20/EC. Both require trial registration prior to its start for clinical trials with medicinal products. A summary of the results must be submitted within one year from the end of the clinical trial, irrespective of the outcome. The Regulation will harmonise the assessment and supervision processes for clinical trials throughout the EU, and is currently scheduled to entry into force in 2019.[22,23] Finally, the Declaration of Helsinki requires all researchers, authors, sponsors, editors and publishers to have ethical obligations with regard to the publication and dissemination of the results of all research.[24]

In the field of otology, the extent of publication bias is still unknown. Therefore, we aim to examine 1) the rate of publication of registered trials in ClinicalTrials.gov, 2) the public availability of the results, including reporting of trial registration numbers, 3) the study characteristics associated with publication, and 4) the time to publication after trial completion.

## Methods

### Search for eligible trials at ClinicalTrials.gov

Trials were obtained from ClinicalTrials.gov on January 10, 2017 by one investigator (JvH). The topic ‘Ear, Nose and Throat Diseases’ was selected and all available information of all registered otorhinolaryngology trials was obtained and exported into a spreadsheet. Subsequently trials with otology topics were extracted, including neuro-otology.

We excluded observational studies, duplicate labelled trials (the same NCT number registered within different topics, e.g. vestibular disease, hearing loss, deafness, etc.), as well as trials with a primary completion date after December 31, 2015, in order to provide at least a one year period in which investigators could submit their results, consistent with FDAAA legislation. All trial statuses (e.g. ‘Completed’, ‘Terminated’, ‘Recruiting’, ‘Unknown’, etc.) and identical trials registered with different NCT numbers were included (e.g. the same trial registered separately by the sponsor and by the principal investigator).

### Search strategy for publication of trial outcomes

**Fig 1:**
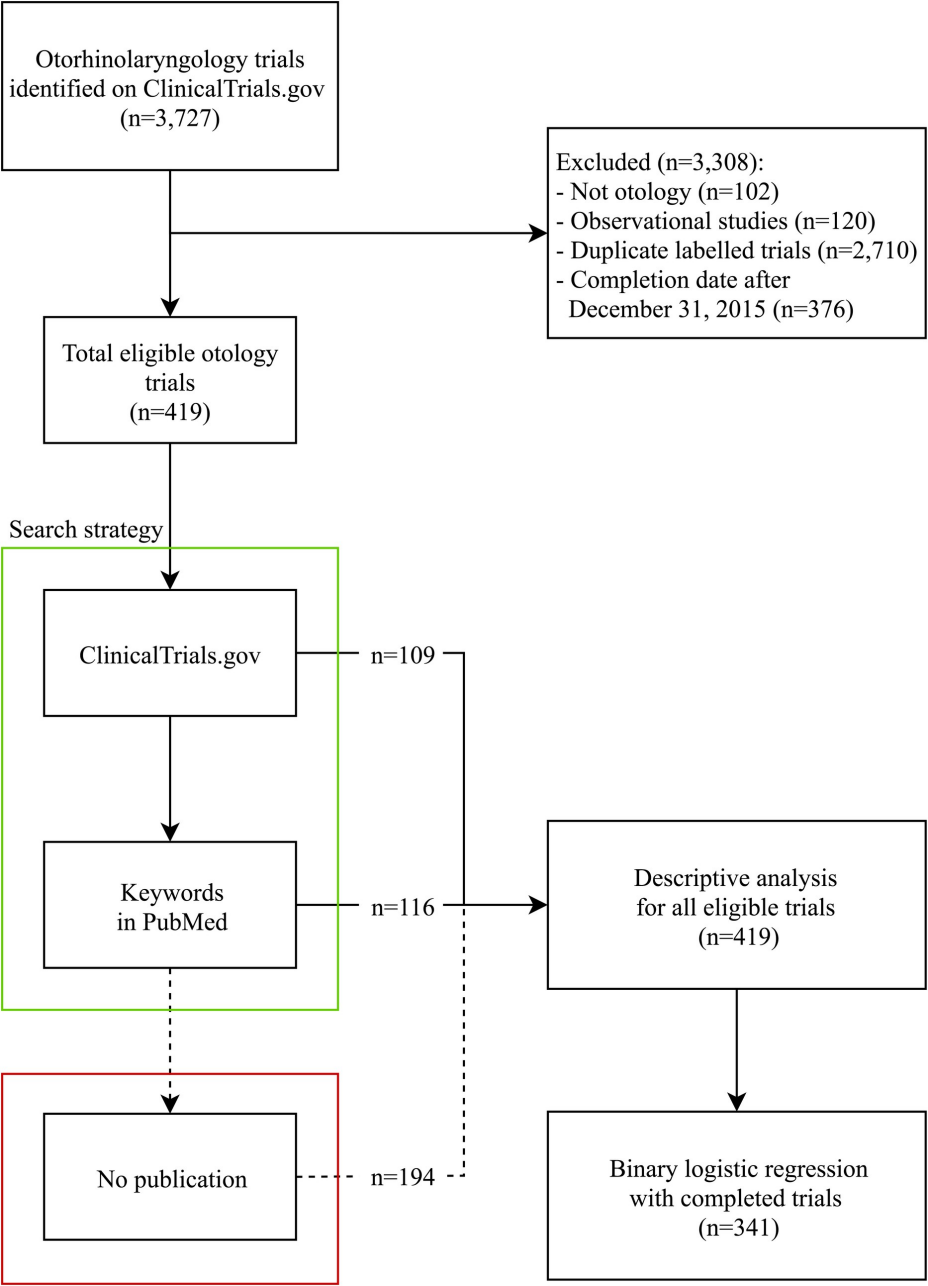
Flowchart of our search strategy and analysis. Our search yielded a total of 3,727 registered otorhinolaryngology trials, of which 3,308 were excluded. In total 419 eligible otology trials were identified.

Two investigators (JvH and IvB) searched for publications of the eligible trials independently using a standardized strategy (Fig 1). If there was any inconsistency, a third investigator (IS) reviewed the data and consensus was reached after discussion. Publications, defined as a complete manuscript in a peer-reviewed journal, were selected up to June 30^th^ 2017, allowing a minimum time of 18 months for the investigators to submit an article for publication, peer review and editorial processes.

1. The ‘more information’ field within ClinicalTrials.gov was examined to determine if the trial sponsor or investigator provided a citation of an article describing the corresponding trial, as this field is used to disclose citations of trial results or other relevant research.
2. If no citation was provided, PubMed was used to search the Medical Literature Analysis and Retrieval System Online (MEDLINE) database for publications. An extensive search was performed using the NCT number, names of (principal) investigators, affiliations/institutions or keywords, e.g. trial title, intervention, primary and secondary outcome measures, study design, etc.

Publications (including preliminary results and pilot studies) of corresponding trials were marked as ‘publication’. If no corresponding article was found, we marked it as ‘no publication’. When results were only reported in ClinicalTrials.gov, we marked it as ‘results reported in ClinicalTrials.gov’. Research protocols and publications that provided the corresponding NCT number, but did not report outcomes of the trial, were excluded. If multiple corresponding publications were found for one trial, the article reporting primary outcome measures was chosen in order to calculate the most accurate time to publication.

### Assessment of independent variables

The following data were extracted from ClinicalTrials.gov: NCT number, recruitment status, type of intervention (e.g. drugs, biological interventions, medical devices, or non-invasive interventions), age of participants, study phase (stages of clinical trials studying a drug or biological product, based on definitions developed by the U.S. Food and Drug Administration (FDA)), study topic, funding, number of patients enrolled, start date, ‘Primary completion date’, ‘Completion date’, and the date ‘Results first received’. In ClinicalTrials.gov the ‘Primary completion date’ is defined as the date that the final participant was examined for the purposes of final collection of data for the primary outcome. The ‘Completion date’ is defined as the date the final participant was examined for the primary and secondary outcome measures, and adverse events.[25] We considered a trial as ‘completed’, if it had one of the following recruitment statuses in ClinicalTrials.gov: ‘Completed, has results’, ‘Completed, no results available’, or ‘Terminated’.

The trial duration was calculated as the number of months from start date to completion date. In our study, the ‘Primary completion date’ was used for this calculation. When this was not provided, the ‘Completion date’ was used. The topic of the selected trials was evaluated by screening the title and the ‘study details’ section in ClinicalTrials.gov.

### Assessment of published articles

Reporting of the NCT number and month and year of publication were noted. Time to publication for published trials was calculated as the number of months from the completion date of the trial to the publication date of the article. The electronic publication date or, when this date was not provided, the publication date in print was used to determine the publication date. Time to publication of results on ClinicalTrials.gov was calculated as the number of months from the completion date of the trial to the date the results were first received.

### Data analysis

Categorizations of data elements were made, with the exception of two continuous variables (patient enrolment and trial duration). The Shapiro-Wilk test was used to test for normal distribution. Patient enrolment and trial duration were not normally distributed.

Age was a continuous variable, which we dichotomized to ‘including children’ (any participants with the age of 0 to 18 years old), or ‘not including children’ (any participants with the age of 18 years old and above). ClinicalTrials.gov classifies funding as follows; industry, NIH, federal government of the United States (U.S. federal), and other (including hospitals, universities, and non-profit organizations). For analysis, we categorized funding to ‘industry’, ‘non-industry’ (including NIH, U.S. federal, and other organizations) or ‘both’, in case several sponsors of multiple funding sources were listed. We categorized interventions to ‘drug’, ‘biological’, ‘device’, and ‘other’ (e.g. behavioral, dietary, genetic, procedure, radiation, and other interventions). When study phase was ‘Not Applicable’ (trials without FDA-defined phases, including trials of devices or behavioral interventions), we classified it as ‘missing’. We dichotomized study topic to ‘tinnitus’ (tinnitus as ‘condition or disease’ with tinnitus outcome measures), or ‘other’ (‘condition or disease’ not including tinnitus). Year of study completion was a continuous variable, which we categorized to ‘before 2006’, ‘2006–2007’, and ‘after 2007’, in order to evaluate the effect of ICMJE requirements (2005) and the FDAAA 801 (2007).

We used descriptive analyses to assess the differences between published and unpublished trials, and time to publication. For the completed trials, the number (percent) for discrete variables and median (interquartile range, IQR) for continuous variables were calculated.

We assessed the possible reasons for publication with binary logistic regression. Univariate regression was used to determine the explanatory variables for the multivariate logistic regression model. Solely variables with p-values less than 0.10 in the univariate level were used. We reported odds ratios (OR) along with 95% confidence intervals (CI) to determine statistical significance for the multivariate regression analysis. We consider a 95% CI not containing 1.00 as statistically significant. In case of discrepancies between p-values and 95% confidence intervals, the confidence interval is leading in the statistical significance. We checked the explanatory variables for multicollinearity. We used IBM SPSS statistics for Windows, version 25.0 (IBM Corp., Armonk, NY, USA) for analysis.

## Results

### Accessibility of results of registered otology trials

**Fig 2:**
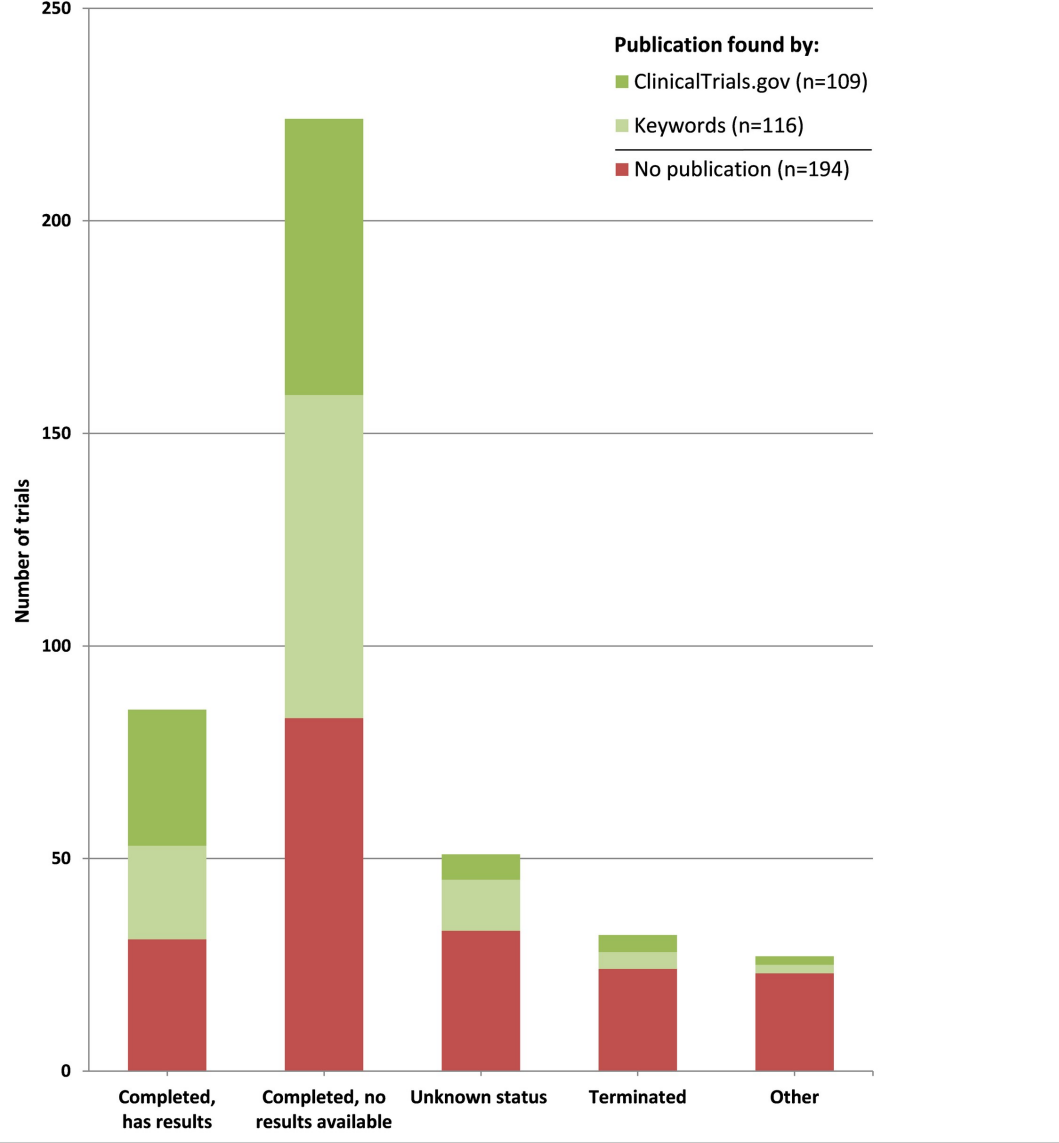
Recruitment status and rate of publication of all otology trials registered in ClinicalTrials.gov. Unknown status: a study on ClinicalTrials.gov which last known status was ‘recruiting’, ‘not yet recruiting’, or ‘active, not recruiting’ but that has passed its completion date, and the status has not been last verified within the past 2 years. Terminated: the study has stopped early and will not start again. Other: trials with recruitment status ‘withdrawn’, ‘suspended’, ‘enrolling by invitation’, ‘recruiting’ and ‘(active,) not yet recruiting’. For all data, see S1 Table.

Our electronic trial registry search in ClinicalTrials.gov yielded a total of 3,727 registered otorhinolaryngology trials, of which 3,308 were excluded (Fig 1). In total 419 eligible otology trials, with start dates from July 1992 to July 2015, were identified (S1 Dataset). The median trial duration was 20 months (IQR 26 months). We found 225 (53.7%) corresponding articles; 109 (48.4%) were reported in ClinicalTrials.gov, and 116 (51.6%) were found on PubMed by keywords (S1 Table and Fig 2). Among all the publications found, 124 (55.1%) reported the NCT number in the article. For 90 (21.5%) of the 419 trials, results were provided in the ClinicalTrials.gov results database. For 36 (40.0%) of these 90 trials, no corresponding peer reviewed articles were found in PubMed. When considering publications and the results reported in ClinicalTrials.gov together, the rate of publication increased from 53.7% to 62.3%. So, in 37.7% of the registered otology trials the results remained unpublished.

Among the 419 eligible trials, 341 (81.4%) trials reported the recruitment status as ‘Completed’ or ‘Terminated’, of which 203 (59.5%) corresponding articles were found. For the remaining 78 (18.6%) trials (e.g. recruitment status ‘Unknown’, ‘Recruiting’, etc.), 22 (28.2%) corresponding articles were found by our extensive search.

### Study characteristics associated with publication of completed trials

Table 1 shows characteristics of completed trials, according to publication status. Trials with a biological intervention were more likely to be published than studies involving drugs (OR 10.41, 95% CI 1.26–86.22, P = 0.030). Trials funded by industry were less likely to be published (OR 0.46, CI 0.25–0.84, P = 0.011). Whether or not children were included, study phase, study topic, year of completion, trial duration, and patient enrolment showed no association with publication (P > 0.05). We found no multicollinearity (Variance Inflation Factor 1.001–1.365) among the explanatory variables.

**Table 1:** Binary logistic regression for effect of different characteristics on publication of completed otology trials registered in ClinicalTrials.gov.

Characteristics		Total (n = 341)	Publication (n = 203)	No publication (n = 138)	Univariate		Multivariate	
		n (%)	n (%)	n (%)	OR (95% CI)	p-value	OR (95% CI)	p-value
Interventions	Drug	134 (39.3)	64 (31.5)	70 (50.7)	Reference		Reference	
	Biological	14 (4.1)	13 (6.4)	1 (0.7)	**14.22 (1.81–111.78)**	**0.012**	**10.41 (1.26–86.22)**	**0.030**
	Device	76 (22.3)	44 (21.7)	32 (23.2)	1.50 (0.85–2.65)	0.159	1.64 (0.89–3.03)	0.117
	Other^a^	116 (34.0)	81 (39.9)	35 (25.4)	**2.53 (1.50–4.27)**	**0.000**	1.73 (0.90–3.33)	0.098
	Missing	1 (0.3)	1 (0.5)	0 (0.0)				
Age	Including children	129 (37.8)	75 (36.9)	54 (39.1)	0.91 (0.58–1.42)	0.683		
	Not including children	212 (62.2)	128 (63.1)	84 (60.9)	Reference			
Study phase	Phase 0	6 (1.8)	3 (1.5)	3 (2.2)	Reference			
	Phase 1	15 (4.4)	9 (4.4)	6 (4.3)	1.50 (0.22–10.07)	0.677		
	Phase 1|2	13 (3.8)	10 (4.9)	3 (2.2)	3.33 (0.43–26.04)	0.251		
	Phase 2	59 (17.3)	36 (17.7)	23 (16.7)	1.57 (0.29–8.43)	0.602		
	Phase 2|3	12 (3.5)	7 (3.4)	5 (3.6)	1.40 (0.20–10.03)	0.738		
	Phase 3	62 (18.2)	29 (14.3)	33 (23.9)	0.88 (0.16–4.70)	0.880		
	Phase 4	28 (8.2)	19 (9.4)	9 (6.5)	2.11 (0.35–12.60)	0.412		
	Missing	146 (42.8)	90 (44.3)	56 (40.6)				
Study topic	Tinnitus	80 (23.5)	53 (26.1)	27 (19.6)	1.45 (0.86–2.45)	0.163		
	Other	261 (76.5)	150 (73.9)	111 (80.4)	Reference			
Year of study completion	Before 2006	37 (10.9)	29 (14.3)	8 (5.8)	Reference		Reference	
	2006–2007	18 (5.3)	13 (6.4)	5 (3.6)	0.72 (0.20–2.62)	0.615	1.50 (0.37–6.11)	0.571
	After 2007	280 (82.1)	158 (77.8)	122 (88.4)	**0.36 (0.16–0.81)**	**0.014**	0.47 (0.19–1.17)	0.106
	Missing	6 (1.8)	3 (1.5)	3 (2.2)				
Funding	Industry	120 (35.2)	53 (26.1)	67 (48.6)	**0.35 (0.22–0.56)**	**0.000**	**0.46 (0.25–0.84)**	**0.011**
	Non-industry	196 (57.5)	136 (67.0)	60 (43.5)	Reference		Reference	
	Both	25 (7.3)	14 (6.9)	11 (8.0)	0.56 (0.24–1.31)	0.181	0.70 (0.27–1.83)	0.461
Trial duration^b^	Median (IQR)	19.5 (25.75)	23.00 (27)	17.00 (23)	**1.03 (1.01–1.04)**	**0.000**	1.02 (1.00–1.03)	0.021
	Missing, n (%)	9 (2.6)	9 (50.0)	9 (50.0)				
Patient enrolment^c^	Median (IQR)	65 (147)	66 (172)	70 (154)	1.00 (1.00–1.00)	0.253		
	Missing, n (%)	6 (1.8)	9 (69.2)	4 (30.8)				

^a^ Behavioral, dietary, genetic, procedure, radiation, and other interventions.

^b^ Calculated as the interval between the start and completion dates, in months.

^c^ Number of patients enrolled in trial.

CI = Confidence Interval; IQR = interquartile range; OR = Odds Ratio

Bold values are statistically significant.

### Time to publication, rate of publication and trial completion over time

**Fig 3:**
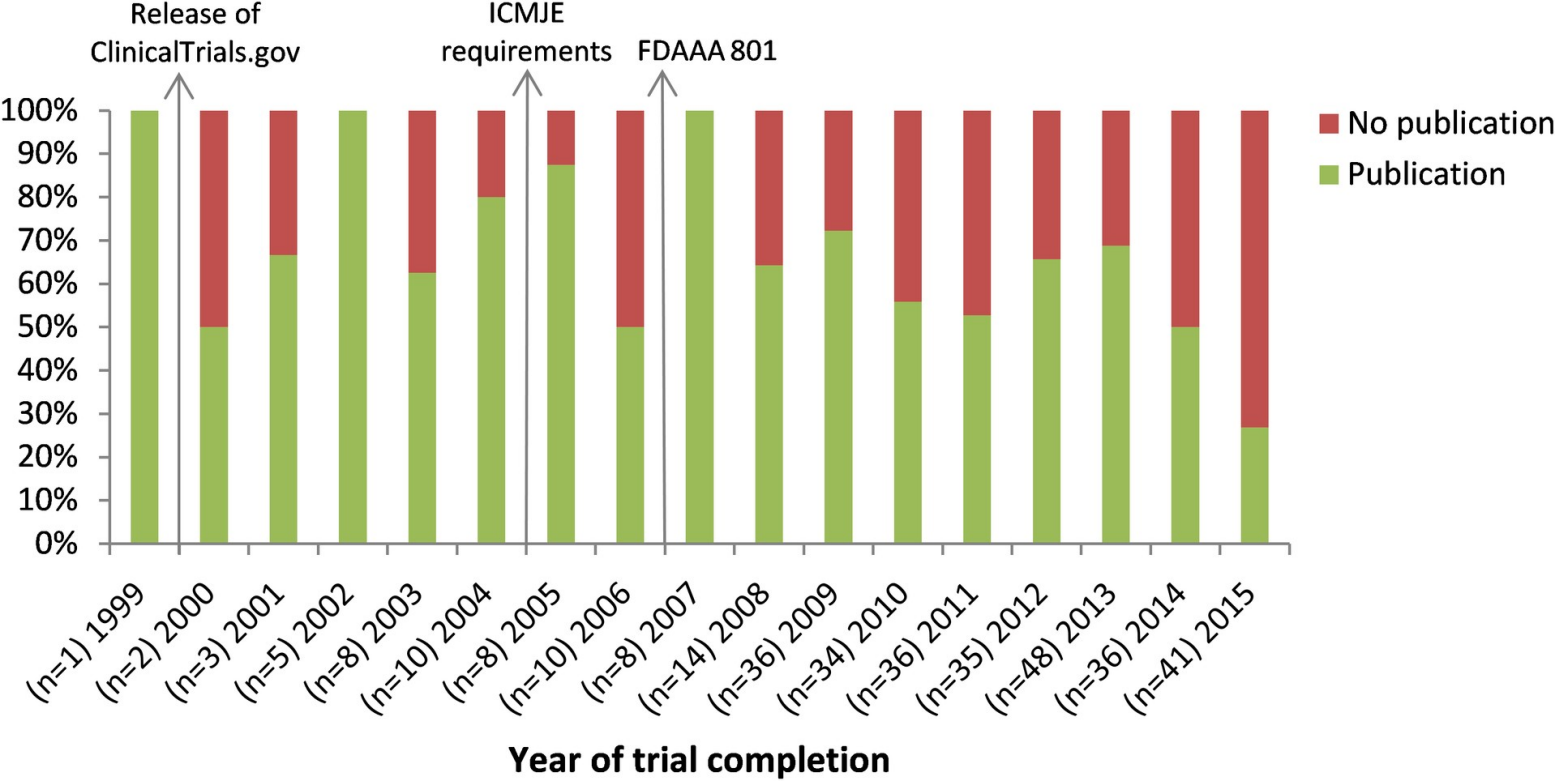
Rate of publication after trial completion over time. Results are shown for 335 completed trials. For six trials, no (primary) completion dates were reported. n = number of completed trials.

**Fig 4:**
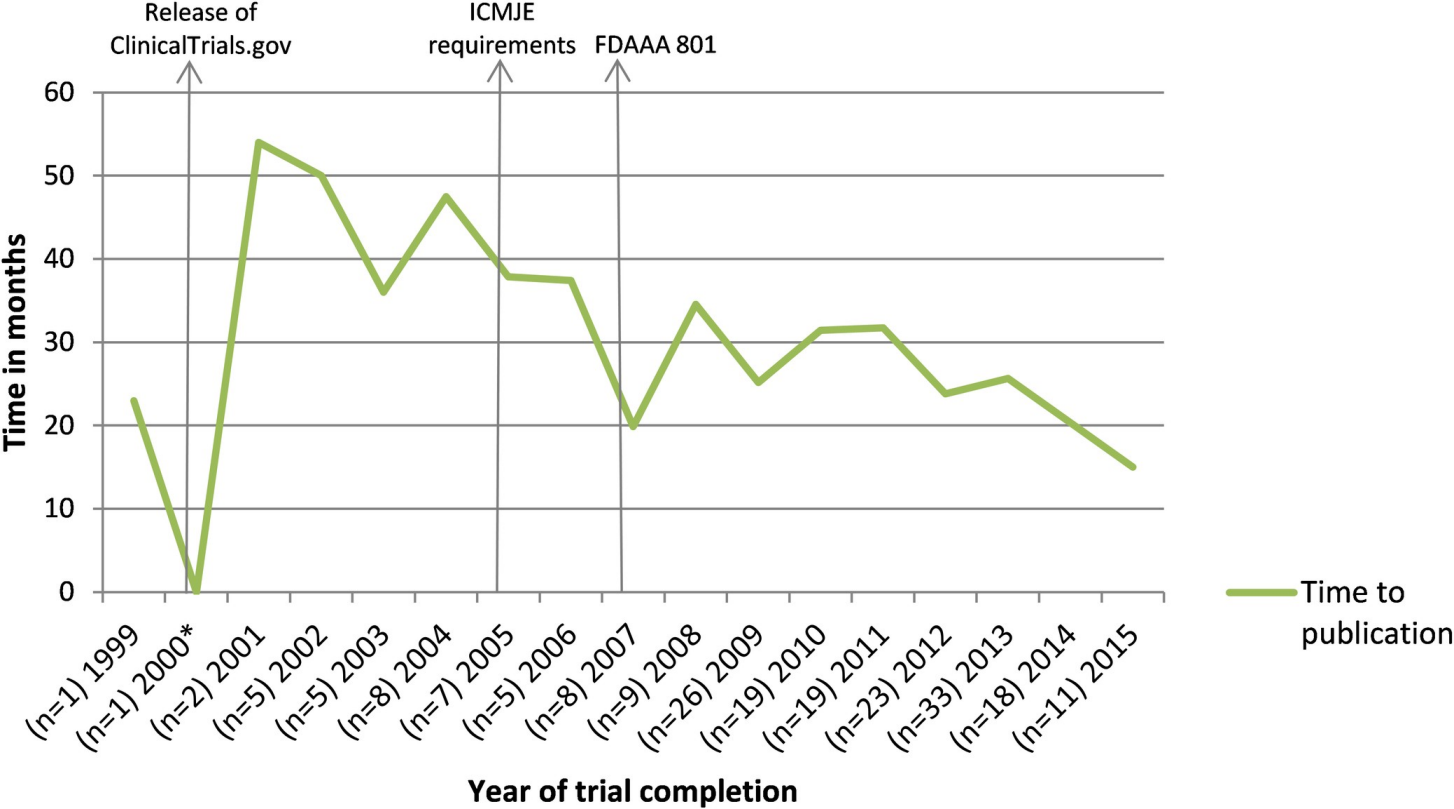
Time to publication after trial completion over time. Results are shown for 200 publications of completed trials. For three published trials, no (primary) completion dates were reported. *Publication before completion date. n = number of completed trials.

The median time from study completion to publication was 24 months (IQR 22 months, n = 200). Among these trials, 21.0% was published within 12 months after study completion. For 90 (21.5%) of the 419 eligible trials, results were reported in ClinicalTrials.gov. For these trials, median time from study completion to ‘results first received’ was 22.5 months (IQR 29 months), and in 27.8% results were published within 12 months after study completion. Fig 3 shows the rate of publication for each year of trial completion. Fig 4 shows a decline in time to publication between 2001 and 2005, which remained constant after 2005.

## Discussion

### Summary of results

This is the first study to assess trial registration, publication rate and characteristics of otology trials. It demonstrated a considerable source of publication bias. In 37.7% of the registered otology trials the results remained unpublished, even several years after trial completion. When publications were found, the majority of the publications was not cited on ClinicalTrials.gov, and a substantial proportion of the publications did not report the NCT number in the article, which would have made it easier for clinicians to access results.

### Comparison with literature

Studies assessing trial registration and publication rate within other fields like rheumatoid arthritis[5], macular degeneration[8], and trials on diseases in the digestive system[7] showed similar outcomes with publication rates of respectively 64.5%, 54% and 63%. Moreover, consistent with other research[5,10,26], our study showed a marginal percentage of completed trials that submitted results in ClinicalTrials.gov. Although trial registry has the potential to improve transparency[27] and to address selective reporting[3], its usefulness is limited by the lack of absolute requirements to complete and update optional data elements, and suboptimal study reporting, as for example the non-reporting of NCT numbers.

Several studies, including ours, showed lower publication rates in trials funded by industry[3,10,11,13], whereas other studies, found no significant association.[5,8,14,28,29] Consistent with our study, a study assessing 143 randomized controlled trials concerning rheumatoid arthritis showed no increase of rate of publication after the ICMJE requirements (2005) or the FDAAA (2007).[5] In contrast to our findings, a cross sectional study of 818 registered surgical randomized controlled trials showed no association of publication with intervention type.[13]

We found a median time to publication of 24 months (IQR 22 months). Consistent with our data, there has been an improvement over the years of publication time reported in literature.[5–7,27] However, we did not take the different lengths of follow-up into account by using survival analysis. This could explain the slight decrease of time to publication after 2014. Therefore, grounded statements on time to publication assessed over time cannot be made.

Studies assessing publication of registered trials did not take every recruitment status into account. It is remarkable that still in 28.2% of trials with recruitment status ‘Unknown’, ‘Recruiting’, ‘Enrolling’, etc. results were published. The majority of these trials had the recruitment status ‘Unknown’: a trial registration that has not been verified by the sponsor or investigator within the past 2 years. This highlights the lack of accurate data submission and registration in ClinicalTrials.gov. In addition, the completion status in trial registries is commonly incorrect.[30] Likewise, we found deficits in registration records of ClinicalTrials.gov; missing and inaccurate data elements, deviations in trial registrations, and deviations in labelling of trial topics.

### Methodological considerations

Our study is the first to assess the trial registration, publication rate, and characteristics of otology trials. We considered all otology trials registered in the largest registry, ClinicalTrials.gov, including trials registered from across the world, operated by the National Library of Medicine at the NIH.[31,32] It permits a detailed assessment of exactly where in the research pathway publication bias can be targeted. We acknowledge that more trials could have been included when other trial registries were used. However, ClinicalTrials.gov provided a representative sample, as it is the largest trial registry by far[33], and thus the publication rate is not likely to be different for other trial registries.

We must consider some limitations of our study. First, only PubMed was searched for publications, which might have induced some omissions, underestimating the rate of publication. However, we have checked that PubMed publishes all otorhinolaryngology journals listed in Journal Citation Reports (JCR), which reduced the probability of omitted publications in our study. Second, the median time to publication in our study transcended the provided 18 months after trial completion to publish the results, meaning that part of the registered trials completed in 2015 might not have been published yet. As a consequence, this could have underestimated the rate of publication and overestimated the probability of publication bias. Finally, investigator bias may have been introduced. The second investigator (IvB) was not blinded for the outcome of the standardized search for publications, conducted by the first investigator (JvH). This may have resolved, subconsciously, in gathering data selectively. However, the risk of bias was reduced by performing the search independently and by a third investigator who reviewed the discrepant data.

## Conclusions

In summary, we showed that otology trails are prone to publication bias. Trials with a biological intervention were more likely to be published than studies involving drugs, and trials funded by industry were less likely to be published. Otology trials are published at a considerably low rate, and time to publication is still extensive. With little citations on ClinicalTrials.gov and low reporting of the National Clinical Trial registry number, the accessibility is limited. Availability of results and traceability of publications and corresponding trials are a clinical and ethical necessity. The benefits of health research can only be realized when study methods and results are submitted or published timely and accurately. Efforts must be made to raise awareness of publication bias and to further enhance accuracy of clinical trial registries, in order to diminish selective publication and non-publication.

## Supporting information

S1 Dataset (XLSX)

S1 Table Recruitment status and search strategy of all otology trials registered in ClinicalTrials.gov. (DOCX)
