# Supplementary material for: Trial registration, publication rate and characteristics in the research field of otology: A cross-sectional study
Source: PLoS One. 2019 Jul 10;14(7):e0219458. doi: 10.1371/journal.pone.0219458 (PMC6619790; doi:10.1371/journal.pone.0219458)
Supplement: S1 Table — (DOCX) [file pone.0219458.s002.docx]

**S2 Table. Recruitment status and search strategy of all otology trials registered in ClinicalTrials.gov**

| **Recruitment status** |  | **No publication** | **Publication:** | 1. Clinical-Trials.gov | 2. Keywords in PubMed |
| --- | --- | --- | --- | --- | --- |
|  | n (%) | n (%) | n (%) |  |  |
| Completed, has results | 85 (20.3) | 31 (36.5) | 54 (63.5) | 32 (37.6) | 22 (25.9) |
| Completed, no results available | 224 (53.5) | 83 (37.1) | 141 (62.9) | 65 (29.0) | 76 (33.9) |
| Unknown status^a^ | 51 (12.2) | 33 (64.7) | 18 (35.3) | 6 (11.8) | 12 (23.5) |
| Recruiting | 3 (0.7) | 3 (100.0) | 0 (0.0) | 0 (0.0) | 0 (0.0) |
| Enrolling by invitation | 2 (0.5) | 1 (50.0) | 1 (50.0) | 0 (0.0) | 1 (50.0) |
| Terminated^b^ | 32 (7.6) | 24 (75.0) | 8 (25.0) | 4 (12.5) | 4 (12.5) |
| Active, not recruiting | 4 (1.0) | 1 (25.0) | 3 (75.0) | 2 (50.0) | 1 (25.0) |
| Withdrawn^c^ | 14 (3.3) | 14 (100.0) | 0 (0.0) | 0 (0.0) | 0 (0.0) |
| Not yet recruiting | 1 (0.2) | 1 (100.0) | 0 (0.0) | 0 (0.0) | 0 (0.0) |
| Suspended^d^ | 3 (0.7) | 3 (100.0) | 0 (0.0) | 0 (0.0) | 0 (0.0) |
| Total | 419 (100.0) | 194 (46.3) | 225 (53.7) | 109 (26.0) | 116 (27.7) |

^a^ A study on ClinicalTrials.gov which last known status was ‘recruiting’; ‘not yet recruiting’; or ‘active, not recruiting’ but that has passed its completion date, and the status has not been last verified within the past 2 years.

^b^ The study has stopped early and will not start again. Participants are no longer being examined or treated.

^c^ The study stopped early, before enrolling its first participant.

^d^ The study has stopped early, but may start again.
